# Heading Towards a Possible Rebirth of the Induced Renal Cell Carcinoma Models?

**DOI:** 10.3390/cancers12030598

**Published:** 2020-03-05

**Authors:** Clarisse R. Mazzola, Domenico Ribatti

**Affiliations:** 1 Department of Urology, University Hospital of Saint-Denis de la Réunion, 97400 Saint-Denis de la Réunion, France; 2 Cancer Registry, University Hospital of Saint-Denis de la Réunion, 97400 Saint-Denis de la Réunion, France; 3 Department of Basic Medical Sciences, Neurosciences and Sensory Organs, University of Bari Medical School, 70124 Bari, Italy; domenico.ribatti@uniba.it

**Keywords:** animal models, kidney cancer, renal carcinogenesis, physiopathology

## Abstract

*Introduction:* Animal models are interesting tools to improve our knowledge of the pathophysiological processes underlying kidney cancer development. Recent advances have been made in the understanding of the genetic founding events underlying clear cell renal carcinoma. The aim of this paper was to review and discuss the characteristics of all the induced animal models of renal carcinogenesis that have been described in the scientific literature to date and to see if and how they could regain some use in the light of the latest discoveries. *Methods:* The authors reviewed all the papers available in PubMed regarding induced animal models of renal carcinogenesis. From this perspective, the keywords “induced”, “animal model”, and “renal cancer” were used in PubMed’s search engine. Another search was done using the keywords “induced”, “animal model”, and “kidney cancer”. PRISMA recommendations were used to develop the literature review. *Results:* Seventy-eight studies were included in this review. Results were presented depending on the mechanisms used to induce carcinogenesis in each model: induction by carcinogens, hormones, viral induction, or induction by other agents. Discussion focused on the possibility to rethink these different induced animal models and use them to answer new research questions. *Conclusion:* Many induced animal models have been developed in the past to study renal cancer. While these models seemed unable to yield new knowledge, the latest advances in the understanding of the genetics behind renal carcinogenesis could well bring the models back to the forefront.

## 1. Introduction

Clear cell renal cell carcinoma (CCRCC) represents 3% of all cancers, with the average age of diagnosis being 65 years [1,2]. The five-year survival of metastatic CCRCC patients is less than 10%, which is the case in about one-third of patients in the diagnosis phase. [1,2]

The propensity of each patient’s CCRCC to metastasize is difficult to predict and prevent, and it is an important therapeutic challenge to investigate, as half of the patients treated for a localized disease will eventually develop a metastatic disease [3,4].

Gerlinger et al. have partly elucidated the key points of the pathogenesis of CCRCC and the development of metastasis [5,6]. They showed that the whole carcinogenesis process begins with initial truncal aberrations along with some branch aberrations [5,6]. They also showed that the process can lead to the concomitant presence of different tumoral subclones in the same patient (intratumoral heterogeneity) with different propensity to metastasize, different spatial distributions, and possibly also different sensibilities to different treatments [5,6].

In the past, different animal models have been used to study CCRCC, including induced and spontaneous animal models. 

The aim of this study was to review the different types of induced animal models of renal carcinogenesis that have been described so far and see how they can contribute to the recent advances in our knowledge of CCRCC pathophysiology.

## 2. Methods

We reviewed all the papers available in PubMed regarding induced animal models of renal carcinogenesis. With this perspective, we used the keywords “induced” AND “animal model” AND “renal cancer” in PubMed’s search engine. Another search was conducted using the keywords “induced” AND “animal model” AND “kidney cancer.” Papers that were duplicates were removed from our analysis, and the ones that were not available for free in full-text or were irrelevant were excluded as well. Additionally, papers written in languages other than English and French were excluded. 

## 3. Results

**Figure 1:**
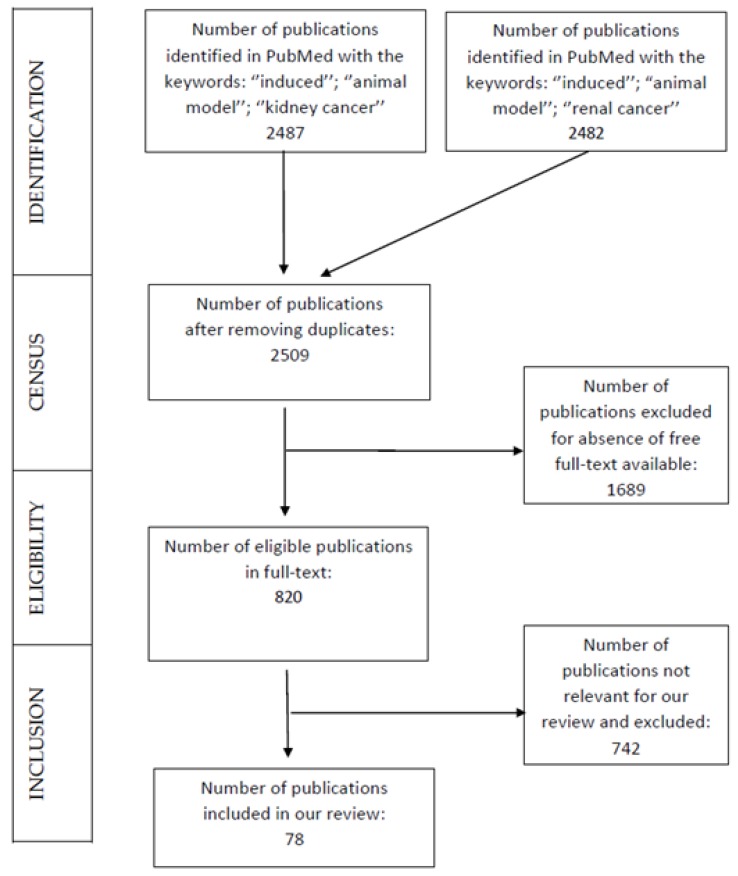
Selection process of the articles in our review following the PRISMA recommendations.

A total of 2487 publications were found in PubMed using the keywords “induced” AND “animal model” AND “kidney cancer”, and 2482 were found using the keywords “induced” AND “animal model” AND “renal cancer.” Both searches retrieved quite similar results. After removing the duplicate publications by pooling the two searches, 2509 publications remained. Among these 2509 publications, 820 free full-text articles could be assessed for eligibility. Of those 820 papers, 742 were removed from the analysis for being irrelevant to our topic—mostly papers dealing with induced animal models of another cancer than kidney cancer (721), and/or being papers on in-vitro models, and/or being papers dealing with non-oncologic kidney diseases—leaving 78 studies to be included in our qualitative synthesis (Figure 1).

## 4. Discussion

Among the numerous induced animal models that were identified in the literature review, three subgroups were seen in terms of how they were induced: by carcinogens, by hormones, and viro-induced. 

### 4.1. Animal Models of CCRCC Induced by Carcinogens

Various chemical agents or heavy metals are known to induce renal cancers in rodents, but the underlying physiopathological mechanisms in their development are poorly understood. Globally, it seems there are two big groups of chemical carcinogens: strong and weak carcinogens [7]. Strong carcinogens such as dimethylnitrosamine (DMN), N-Nitroso-Morpholine (NNM), N-ethyl-N-hydroxyethylnitrosamine (EHEN), or N-(4-∋-Fluro-4-biphenylyl Fluro-4-biphenylyl) acetamide (FBPA) can induce epithelial renal tumors after relatively short latency periods after being either administered in small doses in a single injection or until approximately 10 weeks of exposure through drinking water or food [7]. Weak carcinogens can only induce renal tumors after the administration of strong doses and over longer periods of exposure (generally more than a year) [7,8].

From a chemical point of view, it seems that molecules bearing short alkyl or alkenyl groups (C3 or less) mostly induce tubular adenomas or renal adenocarcinomas. Molecules with amine groups tend rather to induce papillary or medullary renal tumors, or urothelial tumors [8]. Overall, it seems that renal epithelial tumors are the most common kidney tumors induced by chemical carcinogens in rodents [7].

Numerous publications have reported the chemical induction of renal carcinogenesis in laboratory animals. There are probably more than a hundred types of known renal carcinogens [7,8,9]. In this study, the focus will be only on some of the main renal carcinogenesis models that are chemically induced in laboratory animals.

#### 4.1.1. The Animal Models Using Dimethylnitrosamine (DMN) 

The first paper evoking the renal carcinogenic potential of DMN in rats was published in 1960 by Zak et al. [10]. In a later publication by Hard et al., it was shown that a single intraperitoneal injection of 50 to 60 mg/kg DMN administered to five-week-old Porton male rats after 2 weeks of protein-free diet, with only a mixture of glucose and sucrose, induced renal mesenchymal tumors in 81%–100% rats, and renal adenocarcinomas in 19%–31% of the cases [11]. The same observations were made in other studies [12,13].

In a later study by Hard et al., it was shown that the rat age at the time of the exposure to DMN had an important influence on renal carcinogenesis [14,15]. In this study, indeed, the so-induced mesenchymal tumors appeared preferentially in newborn rats or immature rats of 3–4 weeks of age [14,15]. Adult rats aged 9–10 weeks were, on the other hand, more prone to developing epithelial tumors of the renal parenchyma (90% of the cases), which were in 70% of the cases renal adenocarcinomas, mostly in multiples [11,14,15,16].

It seems that the metastases rates described in this model approximated 50% with a preferential localization for lungs [11,14,17].

#### 4.1.2. Animal Models Using N-Nitrosomorpholine (NNM)

In a study published by Bannasch et al. in 1974, it was shown that NNM orally administered at a dose of 12 or 50 mg in drinking water induced all types of renal epithelial tumors in Sprague Dawley rats after oral exposure over short (7–14 weeks) or long periods (until 35 weeks) [18]. These lesions arose in most cases several weeks after NNM intake, with a 69% tumor rate (40/58 rats) between the 22^nd^ and 97^th^ week following the end of treatment [18]. In most of the cases, these tumors were multiple and bilateral [18]. When a single dose of 320 mg/kg was administered orally in one shot, similar results were observed with the occurrence of all types of renal epithelial tumors between 1 and 2 years of age after exposure to the treatment [19]. CCRCC affected 27.3% of rats [19]. Later works by the same group showed that before the development of CCRCC in these animals, a phenomenon of cellular thesaurismose (glycogénose, lipidose, and polysaccharidose) was observed, which could have been caused by the preneoplastic dysregulation of renal cellular metabolism [20]. Mitochondrial anomalies with very wide, round, oval, or sometimes stretched out mitochondria were also observed [21]. A possible disturbance in the metabolism of carbohydrates was proposed by the authors [22].

#### 4.1.3. Animal Models Using Heavy Metals Such as Lead

The first paper evoking the carcinogenic role of lead for rat kidneys was published in 1953 [23]. In a study carried out by Koller et al. in 1985, it was revealed that Sprague Dawley rats exposed to 2600 ppm lead acetate in their water for 76 weeks (chronic lead poisoning) developed renal tubular carcinomas in 81% of the cases (13/16 rats) [24]. In this study, 3/16 rats with tubular carcinoma also had metastases [24]. In a study by Furst et al., the intramuscular carcinogenic effect of lead chromate seemed superior to that of lead in adult male Fischer 344 rats (47/50 rats with cancers against 37/50) with, in decreasing order of frequency, rhabdomyosarcomas (17/50), fibrosarcomas (14/50), renal carcinomas (3/50), and lymphomas (2/50) [25]. In this study, the authors proposed that lead increased the carcinogenic effects of chromates, possibly by the accentuation of porphyrine metabolism within the renal parenchyma [25].

In a later study conducted by Hiasa et al., the role of lead acetate as an amplifier of the renal carcinogenic effects of other chemical substances was demonstrated [26]. In this study, the chronic exposure of adult rats at 20 weeks to 1000 ppm lead acetate after exposure to 1000 ppm EHEN for 2 weeks increased the rate of tumors in the renal tubular system from 50% to 100% compared to rats not exposed to lead acetate [26].

#### 4.1.4. Animal Models Using Streptozotocine

Streptozotocine (a N-nitrosomethylamide) is derived from *Streptomyces achromogenes* [27] and presents diabetogenic [28], antimicrobial [29], and antitumor properties [30]. It is chemically related to dimethylnitrosamine (a N-nitrosodimethylamine), which was described formerly, and is known to have a strong carcinogenic effect on rats [31]. The renal carcinogenic effect on the rat with streptozotocine was described for the first time by Mauer et al. in 1974 [31]. In their study, 30.8% (24/74) of Lewis and Sprague Dawley rats, which lost more than 8 months after the induction of diabetes by streptozotocine, presented macroscopically visible epithelial (tubular and/or papillar) renal tumors, among which two had hepatic and lung metastases [31]. In a study carried out later the same year, the same group showed that the renal carcinogenic effect of streptozotocine was due to the direct effect of streptozotocine on the kidney and not on the diabetic state induced by streptozotocine [32].

In a study published by Horton et al., 36/80 male Wistar rats exposed to a single intravenous dose of 25 mg/kg streptozotocine developed 46 renal tumors, among which 14 were epithelial (similar to adenomas and renal adenocarcinomas in humans), 8 mesenchymal and 24 of mixed appearance (similar to nephroblastomas in humans) [33]. In this study, the diabetic balance had no influence on the incidence of renal tumors, but insulin seemed to speed up tumor growth [33].

In a study by Hillman et al., 4/11 rats developed a renal tumor 6–7 months after the administration of streptozotocine with approximately a total of 12 tumors in these animals [34]. In this study, the spectrum of the observed renal tumors was wide and included both benign as well as malignant renal tumors [34]. Their vascularization was also diversely developed [34].

In an attempt to develop a renal model that could induce renal epithelial tumors with a high frequency, Hard et al. administered 250 mg/kg streptozotocine intravenously to 6-week-old male and female mice [12]. Approximately 73% of males and 97% of females developed renal tumors, but only 31% and 60% of these mice, respectively, presented renal carcinomas [12].

In a study published by Kazumi et al., the carcinogenic effects of streptozotocine, even at a dose of 30 mg/kg, were found to be distinct on the rat pancreas but weak on the kidney [35]. Okawa et al. observed similar results in a population of 16 male Sprague Dawley rats exposed to 60 mg/kg intravenous streptozotocine, with the starting age of 5 weeks and duration of 22 months [36]. In this study, the observed tumors were hepatocellular (69% rats), insulinomas (63%), Leydig cell tumors (56%), kidney adenomas (50%), and cholangiomas (31%) [36]. Similar results were published by Iwase et al. on Wistar Kyoto rats with oncogenetic effects of streptozotocine, essentially on the pancreas, livers, and to a lesser extent on the kidneys [37].

In a study published by Delahunt et al., the renal tumors induced in mice by streptozotocine were highlighted to share pathological similarities with renal chromophobic carcinomas in humans, but with a global ultrastructural morphology differing significantly from that observed in the various pathological renal carcinoma subtypes in humans [38].

In a later study by Gruys et al., the isolation and the use of tumor cells from renal carcinomas induced by streptozotocine in mice were proposed to serve as a tumoral model for the study on renal carcinoma, with a propensity to the development of hypervascularized tumors and lung metastases [39]. In this study, however, the in-vivo tumoral growth after renal implantation of these cells was slow (from 57 to more than 100 days) [39].

Furthermore, in a study by Vinerean et al. performed on Wistar Furth rats exposed to streptozotocine, a major disadvantage of the renal model induced by streptozotocine was identified by a renal tumorogenesis that appeared to be progressive with subsequent modifications through time of the pathology of the so-induced renal tumors [40].

#### 4.1.5. Animal Models Using Urethane

In a study by Vesselinovitch et al., it was shown that MRC rats exposed to urethane (3 or 5 mg in 6 or 10 intraperitoneal injections every 3 days) from the first day of life developed in 7% of cases (11/160) neoplastic lesions of the kidneys [41]. Most of the time, these lesions were voluminous (reaching weights as high as 175 g) and almost always affected the right kidney [41]. These lesions were mostly of embryonic or epithelial types with life expectancies, according to the case, of 62 or 78 weeks, respectively [41]. In 82.5% of the cases, rats presented one or several primitive lesions at the time of death [41]. In a later study by the same authors, it was demonstrated that the neoplastic risk (pathological type, affected organ, and cancer rate) was strongly influenced by the age of exposure to urethane (in utero, 1st, 28th, 46th day of life) [42]. After in-utero exposure, for example, rats had more risk of liver cancers or heart Anitschkow sarcomas, whereas rats exposed later in life developed more gliomas, schwanommas, or embryonic renal cancers [42].

Interestingly, in a study experimenting with Swiss mice, it was seen that male mice born from fathers exposed to urethane did not present more renal cancer risks than control mice [43].

#### 4.1.6. The Animal Models Using Formic Acid 2-(4-(5-Nitro-2-furyl)-2-thiazolyl)-hydrazide (FNT) 

In a study carried out by Ertürk et al. it was shown that formic acid FNT could induce a high frequency of renal carcinomas in Sprague Dawley rats (41.7%; 25/60 rats) and female Buffalo type rats (33.3%; 10/30 rats) [44]. These renal cancers were transplantable without losing their pathologic characteristics and growth speed [44]. In a later study by Hirose et al., factors increasing the carcinogenic potential for the kidney of FNT were studied in a rat population [45]. The incidence of kidney cancer increased markedly when rats were exposed to a combined treatment of chloroform (CHCl3), DMN, FNT, and unilateral nephrectomy (the incidence of kidney cancer reaching 88.9% rats at 32 weeks). It thus seems that the pathogenic effect of FNT might be due to its metabolism by peroxidases [46].

#### 4.1.7. Animal Models Using Methylazoxymethanol Acetate (MAM-Ac) 

In the studies published about rodents, it seems that quite a few carcinogenic effects on the kidney have been reported [47,48]. Some authors have reported the possible occurrence of kidney leiomyosarcomas after intraperitoneal exposition to MAM-Ac in female rats [49]. Others have reported some cases of renal carcinomas (4/10) in African green monkeys after chronic exposure to MAM-Ac (one weekly intraperitoneal injection for 75 months on average) [50]. Some others have reported some renal cancer cases caused by a two-hour MAM-Ac treatment in aquarium fishes [50]. Globally, the MAM-Ac seemed especially carcinogenic for the intestinal tract of rodents [47,48,51,52].

#### 4.1.8. Animal Models Using Iron by-Products 

In a study by Liu et al. Wistar rats repeatedly exposed to ferric diacetate ethylenediamine-N,N′-diacetate [Fe III]-EDDA], an iron chelator, developed a 40% rate of kidney tumors in one year (including kidney adenomas and adenocarcinomas) [53]. The authors proposed that the underlying carcinogenic mechanism rested on the liberation of free radicals [53].

### 4.2. Animal Models of Renal Carcinomas Induced by Hormones

In 1947 and 1952, Matthews, Kirkman, and Bacon studied the various carcinogenic effects of estrogen on the kidneys of male Syrian golden hamsters, following the amount of estrogen delivered, the method of administration, the age of exposure, the type of estrogens delivered, and the existence or not of a surgical castration [54,55,56].

Diethylstilbestrol induced the occurrence of renal tumors in hamsters. However, the administration of concomitant treatment with testosterone, progesterone, or deoxycorticosterone acetate besides diethylstilbestrol inhibited this mechanism of renal tumoral induction [54,55,56].

The renal tumors that were hormone induced in hamsters seemed to arise from the tubular epithelium of the renal cortex [55,56]. They appeared after a subcutaneous injection of 20 mg diethylstilbestrol in hamsters of 6–8 weeks of age. If this only treatment was applied, palpable renal tumors developed in the hamsters in the next 9–12 months. If a second subcutaneous injection of diethylstilbestrol was administered at around 3–4 months of age, this rate increased to 100%. Approximately seven months after the beginning of treatment, the hamsters presented small and pale subcapsular lesions. These lesions then began to increase in number and size and became wide bilateral tumors with hemorrhagic, necrosed, and cystic reorganizations. After 9–10 months, these tumors extended beyond the primitive site. Some researchers also described metastases in lungs and cervical ganglions [57].

These tumors were successfully transplantable to other golden hamsters only if these were also treated with estrogen [58,59,60]. Testosterone increased tumor growth, while progesterone and deoxycorticosterone reduced it [55]. Complete inhibition of tumor growth was attained with the association of cortisone and 6-alpha-methyl-17-alpha-hydroxy-progesterone acetate [61,62]. Surrenalectomy slightly inhibited tumor growth but not as much as a bilateral orchiectomy, which almost totally inhibited tumor development [63]. The administration of estradiol monobenzoate or testosterone propionate restored the tumoral growth inhibited by orchiectomy [61,63].

After more than 5 years of repeated transplantations, the growth of the transplantable renal tumor finally became independent from estrogens [61]. The necessary time-lapse for tumor growth had also considerably decreased: after 9 years of iterative transplantations, a tumor became palpable in the flank of the hamsters in approximately 2 weeks [61].

In later studies by the same group, after more than 95 transplantations of these renal tumoral cells (that had become hormone independent) in golden hamsters, tumor cells could be successfully implanted in cremated hamsters [64]. After three generations of hamsters in which tumor growth was rather slow, tumor growth accelerated to reach that observed in golden hamsters [64]. This growth was not dependent on the administration of exogenous estrogens anymore, but the use of an antiestrogen inhibited tumor growth [64]. The authors thus concluded that the growth of this transplantable tumor remained dependent on endogenous estrogens (derived from suprarenal glands and testicles) [64].

From a pathological point of view, some authors like Horning et al. have suggested that the renal tumors induced by diethylstilbestrol in hamsters were renal adenocarcinomas [58]. In the initial phases of renal carcinogenesis, there was an enlargement of the tubular cell nucleus and a loss of eosinophilia of the cytoplasm of their epithelial cells [55,56,59]. Then the cells progressively duplicated until the tubular lumen was obliterated by a compact mass of cuboïdal, spheroidal, or spindle-shaped cells [55,56,59]. These cells took up coloration a lot and had cytoplasms with unclear limits [55,56,59]. The tubular expansion and growth were followed by the breakage of the basal membrane and the proliferation of epithelial cells between the neighboring tubules [55,56,59]. There was no real encapsulation; tumor growth was made by the peripheral infiltration of adjacent tissues [55,56,59].

Globally, the observed renal tumors looked like human renal tumors in their cystic, hemorrhagic, and yellowish aspect, especially in the case of wide tumors with a cytological aspect of clear cells, papillary architecture, some areas of stellar cells (suggestive of sarcomatous cells), and calcifications [59]. However, unlike what is observed in human renal cell carcinomas, the vacuolated aspect of the clear cells of hamsters seemed to only contain double refractile lipids but no glycogen [59].

### 4.3. Viro-Induced CCRCC Animal Models

In several studies by Prechtel et al., the carcinogenic effect of poliovirus for the kidney of Wistar Lewis rats was demonstrated [60,65]. In these German studies, rats developed quasi-essentially kidney sarcomas with the number, by kidney, size, growth speed, and frequency, directly correlating to the importance of the viral inoculum [60,65]. These results have been reproduced by other authors [66].

It has been shown by some authors that a simultaneous inoculation of the polyomavirus (S.E. form) and of the A/PR 8/34 (HON 1) influenza virus could significantly reduce the rate of kidney sarcomas induced by polyomavirus [67]. The carcinogenic effects on the Wistar rat kidneys could also be observed after the transplacental inoculation of the polyomavirus [67]. These effects, however, seemed to be reduced by the administration of cyclosporine A [67]. All polyomavirus stumps seemed to induce the same sarcomatogenous effects for the kidney [68].

In a study by Corallini et al., it was shown that the BK virus (a polyomavirus from the family of papovavirus) had carcinogenetic properties in immunosuppressed golden Syrian hamsters [69]. The induced tumors were ependymomas, pancreatic carcinomas, lymphomas, osteosarcomas, undifferentiated sarcomas, kidney carcinomas, and the urothelial carcinomas of the renal pelvis, pheochromocytomas and hemangiosarcomas [69]. In a study by Dalrymple et al., 60/78 transgenic mice containing a copy of the BK virus developed renal adenocarcinomas, with significant expression of viral antigens in their kidney cancer specimens and in the thymus of these mice but not in the healthy tissues [70].

### 4.4. Animal Models of Kidney Carcinomas Induced by Other Factors

Many other factors have been used to induce renal carcinogenesis in animals. Among these, we can quote the use of ionizing radiations [71,72,73,74,75] or neutrons [72], or the realization of a single intravenous injection of radioisotopes such as polonium [76]. The induced tumors correspond, as for the renal tumors induced by carcinogens, with adenomas and renal adenocarcinomas [71,72,73,74,75,76]. Finally, other factors seem to accentuate renal carcinogenesis as unilateral nephrectomy [73,77] or chronic unilateral uretero-hydronephrosis (potentialization of the carcinogenic effects of DMN) [78], but these factors do not seem to directly induce the renal carcinogenesis process, strictly speaking.

In a nutshell, animal models of induced renal carcinogenesis have been used for more than about seven decades to improve our understanding of renal cancer pathophysiology. 

Though most of these induced renal cell carcinoma models could seem today somewhat outdated, they have remained very valuable tools to investigate the renal oncogenic potential of various molecules until now. In a study published in 2019 by Hartmann and al., for example, the renal oncogenic effects of angiotensin II were investigated in Big Blue rats [79]. In a study published by Borghoff et al. in 2017, the development of kidney tumors following exposure of rats to ethyl tertiary-butyl ether and tertiary-butyl alcohol was studied as well [80]. In a study by Balansky and al. published in 2018, the carcinogenic response of mice to cigarette smoke was studied at various ages of exposure [81]. There have been some cases, such as in the study of Perry and al., published in 2019, in which long-term combinatorial exposure to trichloroethylene and inorganic arsenic did not induce kidney tumors in genetically heterogeneous mice as it was observed in humans [82], but most of the time, the animal models of induced renal carcinomas have shown to be reliable tools in mimicking the human renal carcinogenesis process.

Besides this known application of the animal models of induced renal carcinogenesis, we believe that they can also yield important pieces of knowledge if they are looked upon from a different angle. Indeed, as these models focus mainly on the renal carcinogenesis process, they constitute a fantastic model to investigate the underlying genetic cascades that lead to renal cancer development and the metastatic process. For example, in the aforementioned animal models of renal carcinogenesis induced by carcinogens, very little is known about the exact mechanisms by which the renal tumors happen following exposure to agents. More specifically the exact sequences of genetic mutations leading to kidney cancer following exposure to all those agents remain mostly unknown, as well as if exposure to one specific agent always leads to the same cascade(s) of mutations or not. In the study by Hartmann and al. published in 2019, it was shown, for example, that exposure to angiotensin II lead to GC → T:A transversions in the transgenic lacI genes of Big Blue rats [79], but in most of the aforementioned models induced by carcinogens especially, the pathophysiology underlying renal carcinogenesis would need to be studied more in depth to allow us to better understand, and maybe prevent, renal cancer development. 

## 5. Conclusions

Many induced animal models have been developed in the past to study renal cancer. While such models seemed unable to yield newer knowledge anymore, the latest advances in the understanding of the genetics behind renal carcinogenesis can put them back on the front stage.
